# Encephaloid Disease of the Antrum, &c by Prof. Hamilton

**Published:** 1857-11

**Authors:** Benj. Heaton Lemon


ART. VII.—*Encephaloid Disease of the Antrum, Ligation of the Primitive Carotid Artery. Removal of the Superior Maxillary and portions of other bones, with the diseased mass.* By Prof. HAMILTON. Reported by BENJ. HEATON LEMON, M. D.

Jan'y 20th, 1856. Mr. A. M., aged 50 years, an intelligent farmer of Smithville, Canada, consulted Prof. Hamilton respecting a "tumor on the right side of his face, and in the roof of his mouth."

On the cheek it was about as large as a medium sized orange, but projected somewhat less in the mouth.

The disease was first observed three or four months ago, but the greater part of its present size has been attained during the last month.

Dr. Hamilton diagnosed cancerous disease of the bone, and advised the speedy extirpation of the parts involved.

Jan'y 25th, the operation was performed, Drs. Boardman, Fitch and Lemon assisting.

Dr. Hamilton observed that there was a considerable increase in the size of the tumor since he first saw it, four days before, and the patient said that for the last two or three days, everything he had eaten seemed to have an offensive taste. On examination the mucous membrane was found abraded with an offensive exudation from the tumor.

The skin over the tumor was sound.

The patient was stripped, placed on a table with his head opposite a good light, and made to inhale a mixture of chloroform and sulphuric ether, until he had fallen into a profound sleep.

Having first ligated the common carotid artery, Dr. Hamilton proceeded to remove the diseased parts. The upper lip was divided by scissors at a point opposite the lateral incision, and the incision continued a little above the wing of the nose.

A second incision was made from the angle of the lower jaw, about two and a-half inches in length, thus making a triangular flap, which, upon being raised and dissected up toward the eye, afforded a good view of the part to be removed. With the bone forceps the jaw was then divided between the lateral incision and the cuspid teeth, and the operation finished with curved chisels; several arteries were cut.

The greater part of the superior *maxillary*, part of the *palatine*, and the lower portion of the *malar* bones were removed with the diseased mass, the soft parts of which were of a brain-like substance.

The anæsthetics were now suspended, and the wounds dressed; he soon regained his senses, and complained very much when the sutures were introduced; he felt no pain during the previous operations. He went on to convalescence, and finally completely recovered, without any accident, and at the present time, twenty months after the operation, I do not learn that there is any symptom of the reäppearance of the disease. I believe he enjoys good health, and is competent to prosecute his ordinary business.

He has, I understand, experienced no ill-effects from the operation, other than a slight defect in speech, but for which, his friends say that they would not know any difference in him.

There is every reason, therefore, for general satisfaction, at the result of so formidable an operation, which by removing a malignant and loathesome disease, has at least prolonged his life, and we are not without encouragement that the malady will never return.

